# Identification of a T1D Susceptibility Gene

**DOI:** 10.1100/tsw.2001.30

**Published:** 2001-05-01

**Authors:** Grant Morahan

**Affiliations:** Complex Genetic Diseases Laboratoty, The Walter and Eliza Hall Institute of Medical Research, PO Royal Melbourne Hospital, Victoria, Australia

**Keywords:** interleukin-12, L-12p40, sib-pair analysis, immunogenetics, insulin-dependent diabetes mellitus (IDDM)

## Abstract

It is not known what causes type 1 diabetes (T1D), which affects over 1 million people in the U.S. alone. Each year, 30,000 young people in the U.S. develop this disease and depend on insulin injections thereafter. Because of the huge cost to the individual, the family, and to society in increased health care costs, it is important to find what makes these people susceptible. The disease process itself is clear: the individual’s immune system — T lymphocytes in particular — attack and destroy the body’s insulin-producing cells. But how and why this autoimmune process starts or proceeds unregulated is still not known.

We are now closer to answering this question, with the description in *Nature Genetics* [1] of the involvement in diabetes susceptibility of a susceptibility gene closely associated with the IL12B gene. This gene is responsible for production of interleukin (IL-) 12p40 [2,3]. IL2p40 is known to influence the course of T cell development, particularly the type of immune mediators those T cells make [4].

Many genes contribute to susceptibility to T1D, but until now there has been wide consensus among geneticists for the identification of only two genes in this disease, and these had been first implicated over 20 years ago: genes of the HLA complex and certain polymorphisms in the promoter of the insulin gene (see Ref. 5 for review). The new finding that the IL12B gene contributes to susceptibility is important as it is known that the T cells promoted by IL12 are responsible for the autoimmune destruction of the insulin- producing cells [5]. Injection of IL12 hastens diabetes in NOD mice [6], a special autoimmune diabetes-prone strain [7].

The recent *Nature Genetics* paper describes a study of 250 pairs of children with diabetes, and showed that one particular variant in the 3′UTR of the IL12B gene was found to be inherited more often than expected by chance. This finding was confirmed in a further sample of over 230 families. Other variants located as close as 13 kb to the 3′UTR polymorphism were not preferentially transmitted to affected sibs. The IL12B variant is actually quite common in the population: its frequency is 0.8 which means that most people will have at least one copy of it. For technical reasons, this high frequency makes it difficult to be detected by conventional genetic analyses. Evidence for involvement of IL12p40 in T1D was strengthened by a novel approach of applying TDT to affected sib pairs based on their linkage status. This approach was based on the logic that the individuals who developed T1D by the action of a particular susceptibility allele were those from families who showed linkage to that allele. Other individuals who do not show linkage may well have become diabetic via other susceptibility genes and so would not be expected to show significant TDT. By applying this simple stratification, it was possible to show that there was a clear difference between the “linked” and “unlinked” families with regard to transmission of IL12B alleles. Finally, cell lines selected based on their IL12B 3UTR genotype showed differences in IL12B expression: the susceptibility allele showed higher expression, which is consistent with expectation from model systems mentioned above.

Identification of a T1D susceptibility gene is important for several reasons. It means that we are closer to understanding the disease process in human T1D, and can now

Contact info: Phone: +61-3-9345-2489; Fax: +61-3-9347-0852
E-mail: morahan@wehl.edu.au
© 2001 with author.

define different subgroups based on IL12B genotypes, as well as different HLA or insulin genotypes. This should make it easier to define other susceptibility genes that operate in the different subgroups. Experimental evidence for the role of IL12 in immune regulation may be relevant to human T1D, and the NOD mouse may serve as a direct model for the disease process that affects at least some people. Finally, there are two wider implications. The approach of investigating allelic transmission based on linkage status requires further evaluation but may be useful for genetic analysis of T1D and other complex genetic diseases. The observation of a high frequency of the T1D susceptibility allele in the population suggests that this allele has been selected for, and is generally beneficial; it is when this allele combines with other susceptibility genes and with as yet unknown environmental stimuli, it results in the autoimmune response leading to beta cell destruction and T1D.

| | | |
|---|---|---|
| Received: | March | 5, 2001 |
| Accepted: | March | 26, 2001 |
| Published: | May | 1, 2001 |
